# Are Fear Campaigns Effective for Increasing Adherence to COVID-Related Mitigation Measures?

**DOI:** 10.1007/s12529-022-10137-y

**Published:** 2022-11-01

**Authors:** Bethany Richmond, Louise Sharpe, Rachel E. Menzies

**Affiliations:** School of Psychology, Faculty of Science, The University of Sydney, NSW, Sydney, 2006 Australia

**Keywords:** COVID-19, Mitigation measures, Fear appeals, Cognitive biases, Health anxiety

## Abstract

**Background:**

Using fear to increase the uptake of preventative health behaviours is a longstanding practice, which could be useful in mitigating the spread of COVID-19. However, the impact of fear campaigns beyond behavioural outcomes has rarely been considered. It is possible that these threatening health messages could heighten health-related anxiety by inducing a tendency to interpret ambiguous stimuli in a threatening manner. This research aimed to evaluate the effects of fear-based articles about COVID-19, on intentions to adhere to mitigation measures and interpretation bias—a core maintenance factor in health anxiety.

**Method:**

Two pilot studies were conducted with the aim of validating our novel COVID-related measures and assessing engagement with the threat manipulation. Following this, 375 community members were recruited through social media for the main study. Participants were then randomly allocated to read an article about COVID which was manipulated on both threat and efficacy. After reading the article, participants then completed measures of interpretation bias and intentions to engage in COVID-19 mitigation measures.

**Results:**

Although the threatening articles consistently produced greater COVID-related threat, they only generated a stronger interpretation bias in the first pilot study. Importantly, threat-based communications failed to enhance intentions to perform mitigation measures in any of the studies. Likewise, reading an article which bolstered self-efficacy did not increase intentions, compared to reading a low efficacy article.

**Conclusion:**

This research suggests that fear appeals are unlikely to increase intentions to perform COVID-related mitigation measures.

**Supplementary Information:**

The online version contains supplementary material available at 10.1007/s12529-022-10137-y.

## Introduction

Since its first appearance in Wuhan, China, in December 2019, the novel coronavirus (COVID-19) has spread exponentially. This culminated in a pandemic being declared by the World Health Organisation in March 2020 [1]. In addition to being highly contagious, COVID-19 has caused the deaths of more than 6 million people [2], included in this is the deaths of more than 7900 Australians [3]. Early in the pandemic, mitigation measures were the only way to “flatten the curve” of new cases [4] and ensure that hospitals can meet demands for essential resources, such as ventilators and ICU beds [5]. Since then, effective vaccines have been developed and distributed internationally. However, with the emergence of the more vaccine-resistant Omicron variant [6], mitigation measures remain an essential strategy in minimising the spread of COVID-19. Unfortunately, as the pandemic has persisted and the availability of vaccines has ushered in a sense of complacency [7], overall compliance has waned [8–10] and with that, case numbers have increased exponentially [2].

One way to encourage continued adherence may be using a ‘fear appeal’. Fear appeals are messages which are designed to incite fear about a particular health-threat, to foster changes in one’s health behaviours [11, 12]. However, according to Witte’s Extended Parallel Process Model [13] (EPPM), fear appeals are only effective when both threat and efficacy appraisals are high. Perceived threat is assessed first and is determined by judgements of the lethality of the threat (i.e. *severity*) and the degree of vulnerability to the threat (i.e. *susceptibility*) [14, 15]. If the threat satisfies both criteria, then fear will be experienced. Without sufficient fear, an appraisal of the need to act would not eventuate, resulting in a lack of response to the fear appeal [13]. However, for behaviour change to occur, in addition to feeling highly threatened, an individual must also evaluate the recommended health behaviour as being both effective (i.e. *response efficacy*) and feasible to implement (i.e. *self-efficacy*) [13]. In support of this, meta-analyses of the last 60 years of fear appeal research have found that the persuasiveness of threatening messages is enhanced through their combination with efficacy-boosting components [16–19].

Threat and efficacy perceptions have also been identified as key predictors of the uptake of mitigation measures during disease outbreaks. Specifically, studies conducted during the severe acute respiratory syndrome (SARS) outbreak in 2003 found that greater anxiety and risk perceptions were associated with uptake of health-protective behaviours [20–22]. Comparably, research conducted during the 2009 H1N1 (swine flu) pandemic indicated that stronger efficacy and threat appraisals were associated with better adherence to [23], or stronger intentions towards, pandemic flu mitigation measures [24, 25]. Additionally, it was found that these appraisals could be successfully manipulated via health messages [24, 25]. Perhaps, most relevant is the research conducted during the COVID-19 pandemic, which has found that greater uptake of mitigation measures is related to heightened risk appraisals of COVID [26–30], greater COVID-related worry [31–33] and fear [34]. Similarly, studies conducted during the COVID-19 pandemic have also supported the instrumental role of response and self-efficacy in the uptake of mitigation measures [35–37].

Previous research has also demonstrated that enhancing efficacy is more influential upon outcomes than threat [16, 38, 39] and can elicit behaviour change without the need for significant threat [40, 41]. This is important as both the EPPM [13] and empirical research suggests that in the absence of high efficacy, threatening messages can be counterproductive [18, 39, 42]. Aligned with this, research conducted during the COVID-19 pandemic found that not only were response and self-efficacy the strongest predictors of adherence to mitigation measures, but that stronger threat appraisals of COVID-19 were at times associated with greater non-compliance [35]. Thus, it is an imperative that research is directed towards assessing whether the impact of fear appeals upon intentions to perform COVID-19 mitigation measures.

To date, just one study has assessed the impact of a COVID fear appeal. Heffner et al. [43] had participants read either a threatening message which emphasised the severity of COVID and participant’s susceptibility to it, or a prosocial message which instead focused on increasing self and response efficacy towards mitigation measures. They found no difference in the effectiveness of these messages in encouraging uptake of mitigation measures. However, they did find that participant’s emotional responses to the messages differed, with the threatening message eliciting a stronger and more negative emotional response in comparison to the prosocial message. Importantly, the persuasiveness of the threatening message was not dependent upon the emotional reaction it elicited. Ultimately, these findings lend support to the idea that fear appeals may be unnecessarily intensifying negative emotions, without any benefits in adherence. Nevertheless, as this study did not independently manipulate threat and efficacy, it is unclear whether increasing threat alongside efficacy would produce adaptive changes in intentions to adhere to COVID mitigation measures.

In addition to this, there is a need for additional research to investigate the cognitive processes which may be underlying emotional reactions to these threatening messages and maintaining fear and anxiety in the long term. Unlike the pandemics of the past, COVID-19 is the first to take place in a world where information is highly accessible due to social media and 24-h news cycles. As such, there has been no shortage of alarmist news pieces about COVID. Within this context, it is especially important to consider what impact fear appeals may have beyond increasing adherence to mitigation measures. Equally as important as promoting adherence is safeguarding the mental health of the vulnerable individuals within our communities. Crucially, within a pandemic, those with elevated health anxiety may be highly vulnerable to a worsening of their anxiety and unhelpful behaviours, such as excessively seeking reassurance from doctors or panic buying—both of which place additional strain on resources and systems which are already overburdened [44, 45]. Alternately, those high in health anxiety may instead implement avoidance behaviours, preferring to cancel necessary medical appointments for fear of contracting disease [44, 46].

One of the ways in which threatening COVID-related health messages could induce or exacerbate anxiety is by instilling an interpretation bias towards threat. According to the Cognitive Behavioural Model of Health Anxiety (CBMHA), it is the interpretation of ambiguous sensations as threatening that maintains health anxiety, as it leads people to focus on their somatic symptoms, which in turn increases their anxiety [47]. The CBMHA also proposes that these biases can be elicited transiently, or be exacerbated by ‘a critical incident’, during which concern about a health threat is heightened [47]. It is possible that exposure to a fear appeal may act as a ‘critical incident’, biasing participants to interpret future information as threatening, and maintaining their anxiety in the long term. Therefore, this implicit interpretation bias is a possible mechanism through which fear campaigns could exacerbate anxiety. Yet, the impact of fear campaigns upon this process has never been studied.

### The Present Studies

This research aims to evaluate the utility of fear appeals, by assessing their impact upon interpretation bias and intentions to adhere to COVID-19 mitigation measures. As such, this research will elucidate how to best communicate about the COVID-19 pandemic. To achieve this, two smaller pilot studies were conducted in student samples, followed by a large and well-powered study which recruited female participants from the community. The first pilot study was designed to validate a novel COVID-19 scale designed for this study and pilot our threat manipulation. The second pilot study sought to assess participant’s engagement with the articles in the online setting, by assessing memory for facts featured in the article. In our final study, participants were randomly allocated to read an article about COVID which was manipulated on threat and efficacy, before completing measures of interpretation bias and intentions to perform mitigation measures.

Consistent with past research which has found that increasing threat or efficacy positively impacts upon health-related behaviours, we expected that:

H1. Reading a threatening article would strengthen intentions to adhere to mitigation measures.

H2. Intentions would be higher after reading an efficacy-boosting article.

In addition to this:

H3. Aligned with the EPPM, we hypothesised that there would be an interaction effect between efficacy and threat, such that the threatening message would be more effective when combined with the high-efficacy manipulation.

H4. Consistent with the CBMHA, we expected that the threatening article would elicit greater COVID-related threat and a stronger health-threat interpretation bias.

## Method

### Pilot Study 1

#### Participants

Ninety participants (66 males and 24 females) took part in the first pilot study (80 undergraduate students and 10 community members recruited via social media), the age of which ranged from 18 to 75 years (*M* = 22.93, *SD* = 9.84). We manipulated threat (high threat vs. low threat) as the between-subjects variable. The dependent variables of interest were interpretation bias, and intentions to adhere to COVID-19 mitigation measures. This study was approved by The University of Sydney's Human Research Ethics Committee.

#### Measures

##### Demographics

Seven questions measured demographics, including whether participants had any health conditions making them more susceptible to COVID-19, whether they resided with another with a susceptibility to COVID and current employment status (specifically whether participants were essential workers who may be at greater risk).

##### The Short Health Anxiety Inventory

The Short Health Anxiety Inventory (SHAI) [48] consisted of 14 items assessing health anxiety, where higher scores indicated greater health anxiety. For every item, participants selected one of the four provided statements which best applied to them (e.g. If I hear about an illness I never think I have it myself), which were scored from 0 to 3 [48]. The SHAI demonstrated good internal consistency (α = 0.88).

##### The Revised Death Anxiety Scale

The Revised Death Anxiety Scale (RDAS) [49] is a 25-item scale which measures fears associated with death. The RDAS can be presented either in either true or false form or using a 5-point scale [49]. We opted for the 5-point scale to allow for greater variability in responses. As such, participants indicated the extent to which they agreed with each statement (e.g. I fear dying a painful death) and responses were scored from 1 (*Strongly Disagree*) to 5 (*Strongly Agree*), or 5 (*Strongly Disagree*) to 1 (*Strongly Agree*), for the reverse coded items—where higher scores indicated greater death anxiety [49]. The RDAS demonstrated excellent internal consistency (α = 0.91).

##### COVID-19 Adherence to Mitigation Measures Scales

The COVID-19 Adherence to Mitigation Measures Scales (CAMMS) consisted of four sub-scales, each consisting of ten questions, with a total of 40 items. One measured past adherence to mitigation measures (e.g. ‘I always wash my hands thoroughly’) and the three others measuring the Theory of Planned Behaviour [50] variables: Attitudes (e.g. ‘Coughing into a tissue or my elbow is important’), Subjective Norms (e.g. ‘Most people avoid touching their face’) and Intentions to adhere to COVID-19 mitigation measures (e.g. ‘I will not shake other peoples’ hands’). There were 10 mitigation measures included in these scales, which were drawn from the hygiene and physical distancing guidelines, published by the Australian Government Department of Health [51, 52]. For every item, participants responded on a 7-point scale, indicating the extent to which they agreed with the statement. Scores ranged from 1 (*Strongly Disagree*) to 7 (*Strongly Agree*), with higher scores indicating stronger agreement. All of the sub-scales demonstrated sufficient internal consistency (see Table 1).

**Table 1:** Study 1 internal consistency reliability of novel measures

Measure	Cronbach’s alpha	Assessment
Past adherence	.74	Acceptable
Attitudes	.84	Good
Subjective norms	.83	Good
Behavioural intentions	.86	Good

##### Manipulation Check

Three items were designed to assess whether the articles manipulated COVID-related threat. Each item consisted of a statement about COVID-19 (e.g. ‘I am worried about COVID-19’) and participants indicated whether they agreed with the statement. Responses were scored from 1 (*Strongly Disagree*) to 7 (*Strongly Agree*), where higher scores indicated greater COVID-related threat.

##### Interpretation Bias

The ambiguous word task [53] is a previously validated measure of interpretation bias. It is comprised of 14 ambiguous words, which participants were instructed to respond to with the first word that came to their mind. Responses were then coded as either being health threat-related or neutral.

#### Materials

##### The News Articles

We chose to use a mock news article as a vehicle for the manipulation of threat (and later efficacy). We chose an article format for its ecological validity, given that at the time we commenced this study, daily reports were being circulated across news media with threatening information about COVID-19. Hence, the impact of threat being manipulated through written media was of primary interest. All participants read an article which included information regarding the prevalence and severity of COVID-19. Both articles were matched for length and contained the same information, with the statistics included updated on the day recruitment began (18 May 2020), using the website https://www.worldometers.info/coronavirus/. There were two versions of the article: One which was written with a threatening tone and the other with a reassuring tone. See appendix for the articles.

#### Procedure

Recruitment for the first pilot study was undertaken between the 18th and the 29th of May. In the preceding week, there had been 104 new cases of COVID-19 in Australia [3]. At this time, restrictions had only been slightly eased following a nation-wide lockdown of almost 2 months [54], with 5–10 household visitors allowed, and some states permitting restaurants to open at a limited capacity [55].

Participants accessed the information statement and consent form online. Upon giving consent, they then completed measures of health anxiety, death anxiety, past adherence, attitudes and subjective norms. Participants were then randomized electronically to the threatening or reassuring condition and were instructed to read the presented article. Following this, they filled out the manipulation check, before completing the measures of interpretation bias and behavioural intentions.

#### Brief Results of the Pilot Study 1

The highly threatening article was successful in producing greater COVID-related threat (*M* = 18.04, 95% CI = [17.38, 18.71]), relative to the less threatening article (*M* = 15.82, 95% CI = [14.90, 16.74]), *t*(88) = 3.97, *p* < 0.001, 95% CI for the mean difference = [1.11, 3.34], Cohen’s *d* = 0.84. Behavioural intentions did not differ significantly after reading the high threat article (*M* = 61.33, 95% CI = [59.28, 63.37]) relative to the low threat article (*M* = 58.86, 95% CI = [56.33, 61.40]), but the difference was in the predicted direction, *t*(88) = 1.53, *p* = 0.129, 95% CI for the mean difference = [−0.73, 5.66], Cohen’s *d* = 0.32. The difference in intentions constituted a small effect, and it is likely that the pilot study was under-powered to detect this effect.

Lastly, participants who read the high threat article made significantly more health-threat responses (*M* = 2.46, 95% CI = [2.09, 2.83]), relative to those who read the low threat article (*M* = 1.77, 95% CI = [1.32, 2.21]), *t*(87) = 2.42, *p* = 0.018, 95% CI for the mean difference = [0.12, 1.26], Cohen’s *d* = 0.51.

#### Decisions for the main study

The first pilot study aimed to validate our novel CAMMS measures, to determine whether these should be implemented in our main study. As the CAMMS measures all demonstrated acceptable internal consistency, they were adopted for the main study. In addition to this, as the threat manipulation successfully increased threat, and elicited a stronger interpretation bias, this was also implemented in the main study.

### Pilot Study 2

#### Participants and Design

A total of 187 undergraduate students (123 females and 64 males), the age of which ranged from 18 to 31 years (*M* = 20.10, *SD* = 2.21), were recruited for the second pilot study. This study was approved by The University of Sydney's Human Research Ethics Committee. Threat (high threat vs. control) was manipulated as the between-subjects variable. The dependent variables of interest were recognition of COVID facts featured in the threatening article, interpretation bias and intentions to adhere to COVID-19 mitigation measures.

#### Measures

As in Pilot Study 1. See Table 2 for internal consistency of measures.

**Table 2:** Internal consistency of novel measures in study 2

Measure	Cronbach’s alpha	Assessment
Death anxiety	.80	Good
Health anxiety	.86	Good
Past behaviours	.82	Good
Attitudes	.85	Good
Subjective norms	.88	Good
Behavioural intentions	.86	Good

##### Memory Measure

The novel memory measure assessed participant recognition of COVID-related facts featured in the threatening article using 14 multiple choice items, which offered one correct option and three distractors. Better memory for COVID-related facts was indicated by participants correctly recognising a greater proportion of threatening information relative to reassuring information.

#### Materials

The same highly threatening article from Pilot Study 1 was used. However, to ascertain whether participants were reading the articles, we replaced the less threatening article with a control article about pandas. Both articles were matched for length and the COVID-related statistics in the threatening article were updated on the day recruitment began (21st of October, 2020), using the same website.

#### Procedure

The procedure was identical to the first pilot study; however, we also administered the memory measure at the end of the study. Recruitment occurred from the 21st of October, until the 20th of November. In the week prior, there had been 127 new COVID cases [3]. Victoria (a state which borders New South Wales, in which the study was conducted) was still in a strict lockdown; however, mid-way through, the study restrictions were eased and two visitors were allowed at households [56]. In the remainder of Australia, restrictions were relatively relaxed, allowing at least 20 visitors to households and the majority of businesses to open—albeit some at a reduced capacity [57].

#### Brief Results of Pilot Study 2

Consistent with the first study, participants who read the threatening article had significantly greater COVID-related threat (*M* = 7.67, 95% CI = [7.05, 8.29]), compared to those who read the control article (*M* = 6.75, 95% CI = [6.11, 7.38]), *F*(1,180) = 4.04, *p* = 0.046); however, the effect size was considerably smaller Cohen’s *d* = 0.30. Behavioural intentions were not significantly different between those who read the threatening article (*M* = 23.36, 95% CI = [21.37, 25.34]) and those who read the control article (*M* = 25.21, 95% CI = [23.18, 27.23]), *F*(1,180) = 1.60, *p* = 0.207, η_p_^2^ = 0.009.

Those who read the threatening COVID article correctly recognised a significantly greater proportion of information (*M* = 0.42, 95% CI = [0.39, 0.45]) compared to those who read the control article (*M* = 0.31, 95% CI = [0.28, 0.34]), *F*(1,180) = 22.39, *p* < 0.001, η η_p_^2^=0.111. This suggested that despite the online design, participants were reading and engaging with the article.

Finally, in contrast to the results of the previous study, there was no significant difference in the number of health-related responses made by participants who read a threatening article which did not have a stronger interpretation bias (*M* = 4.01, 95% CI = [3.65, 4.38]), relative to those who read the control article (*M* = 4.18, 95% CI = [3.80, 4.55]), *F*(1,180) = 0.38, *p* = 0.537, η_p_^2^ = 0.002.

#### Decisions for the Main Study

The second pilot study was successful in alleviating concerns about the impact of the threat manipulation in an online setting and when competing with so much COVID-related media at the time. In fact, we found that participants who read the highly threatening articles not only reported stronger COVID threat perceptions, but also demonstrated enhanced recognition of the information featured in the article. As such, we felt confident that our threat manipulation was successful even when administered in an online setting. To reduce participant burden and maximise participant numbers, our memory measure was omitted from the final study. Additionally, a more specific measure of COVID-related health anxiety was chosen for the main study.

### Main Study

#### Participants

Participants were eligible for the study provided they were over the age of 18 and were currently residing in Australia. Five hundred and thirty-seven participants were recruited from the community by sharing an advertisement across the research team’s Twitter and Facebook pages. Of these, 11 were not eligible: two were under the age of 18, and nine did not reside in Australia. Of the remainder, 129 participants did not complete any of the dependent variables (75% completion rate), and one participant failed an attention check. As just 21 of the remaining 396 participants were male (*n* = 16) or non-binary identifying (*n* = 5), we decided to exclude them. Importantly, we ran all analyses both with and without these participants and found that this had no bearing on the results. This left us with a final sample of 375 female participants, whose ages ranged from 18 to 76 years (*M* = 31.67, *SD* = 10.50). This study was approved by The University of Sydney's Human Research Ethics Committee.

#### Design

This study consisted of a 2 (Threat: High, Low) × 2 (Efficacy: High, Low) between-subjects research design. The dependent variables were interpretation bias and intentions to adhere to COVID-19 mitigation measures.

#### Measures

The same measures were used from the previous pilot studies, with the following exceptions. See Table 3 for internal consistency of measures.

**Table 3:** Study 3 internal consistency reliability of measures

Measure	Cronbach’s alpha	Assessment
Death anxiety	.91	Excellent
Past behaviours	.79	Acceptable
Attitudes	.89	Good
Subjective norms	.87	Good
Behavioural intentions	.88	Good

##### The COVID Stress Scales

The COVID Stress Scales (CSS) [58] replaced the SHAI. The CSS are a newly developed scale consisting of 36-items which form 5 subscales. We utilised the three most relevant sub-scales: COVID danger and contamination fears (e.g. I am worried about catching the virus), COVID traumatic stress symptoms (e.g. I had bad dreams about the virus) and COVID compulsive checking and reassurance seeking (e.g. Searched the Internet for treatments for COVID-19), to create a measure of COVID-related anxiety. Responses were scored on a 5-point scale, where higher scores indicated greater anxiety. Our measure of COVID-related anxiety, comprised of these three subscales, demonstrated acceptable internal consistency (α = 0.79).

##### Manipulation Checks

In addition to the manipulation check used in the pilot studies, perceived efficacy was also measured using three items. Each item consisted of a statement, (e.g. ‘I feel capable of reducing my risk of getting infected with COVID-19’) which measured self- or response efficacy. Participants indicated on a 7-point scale the extent to which they agreed with the statement, with scores ranging from 1 (*Strongly Disagree*) to 7 (*Strongly Agree*). Higher scores indicated greater perceived efficacy.

#### Materials

##### The News Articles

The same threat manipulation used in the pilot studies was implemented in the main study, with a few key changes. Firstly, the articles were also updated to discuss the easing restrictions and the possibility of a second wave—as these were pressing issues at the time. In addition to this, the statistics used in the articles were updated on the day recruitment began (on the 7th of July) using the same website. Lastly, we added a new efficacy manipulation, to better align with the EPPM. Efficacy was manipulated by presenting the mitigation measures either as highly effective and feasible, or as being potentially unnecessary and difficult to adhere to.

#### Procedure

The procedure was identical to that used in the pilot studies, apart from the previously outlined changes. Recruitment took place from the 7th to the 9th of July. At this time, restrictions had been significantly eased in all Australian states, except Victoria, allowing at least 20 household visitors [59]. In addition to this, restaurants, gyms, cinemas, and beauty salons had reopened across most States [59]. However, 819 new COVID-19 cases had been reported in Australia during the week prior [3], which resulted in several Melbourne postcodes being put into lockdown [60].

#### Data Analysis Plan

The data was analysed using IBM SPSS software (version 27). To test that randomisation was successful, two-way analyses of variances (ANOVAs) and chi-square tests were conducted on baseline, demographic and medical variables. Any significant differences on these variables resulted in them being entered as covariates in subsequent analyses. To test whether the manipulation was successful, two-way ANOVAs were conducted on the threat and efficacy measures. Two-way ANOVAs were also conducted on the number of health-threat responses made in the ambiguous word task and on behavioural intentions. Lastly, Pearson correlations were calculated between the baseline and dependent variables.

## Results

### Preliminary Analyses

Baseline COVID-related anxiety was significantly higher in those who read the low threat article, *F*(1,371) = 5.53, *p* = 0.019, and subjective norms were higher amongst those who read the high threat article, *F*(1,371) = 4.18, *p* = 0.042 (see Table 4 for means). Additionally, the low threat condition contained a greater number of participants who were susceptible to COVID-19 (*n* = 32), relative to the high threat condition (*n* = 16), χ^2^(1) = 5.64, *p* = 0.018. As such, COVID-related anxiety, subjective norms and COVID susceptibility were entered as covariates in subsequent analyses, in order to have a cautious approach to analyses.

**Table 4:** Baseline and dependent variables means and standard deviations by condition

Variable	High threat (*n* = 185)		Low threat (*n* = 190)	
	High efficacy (*n* = 91)	Low efficacy (*n* = 94)	High efficacy (*n* = 96)	Low efficacy (*n* = 94)
**Baseline variables**				
Age	31.18 (10.05)	31.61 (10.95)	31.00 (8.89)	32.88 (11.95)
COVID-related anxiety	23.92 (15.01)	23.88 (14.29)	27.55 (15.47)	27.45 (14.43)
Death anxiety	76.41 (17.79)	75.36 (15.28)	76.18 (16.30)	79.16 (18.46)
Adherence	50.60 (9.15)	51.29 (9.74)	50.81 (9.12)	50.09 (9.53)
Attitudes	61.66 (7.38)	61.39 (8.56)	61.94 (7.66)	61.68 (8.57)
Subjective norms	38.99 (9.92)	38.43 (9.51)	36.29 (10.28)	36.91 (10.12)
**Dependent variables**				
Threat	17.04 (3.46)	16.69 (3.57)	16.39 (3.70)	16.67 (3.13)
Self-efficacy	6.07 (0.95)	5.71 (1.18)	5.97 (0.92)	5.79 (1.23)
Interpretation bias	3.81 (1.87)	4.22 (2.31)	4.16 (1.99)	4.03 (2.03)
Behavioural Intentions	57.82 (8.85)	58.16 (9.83)	58.23 (8.98)	58.01 (9.78)

### Main Analyses

Participants in the high threat condition had significantly greater COVID-related threat (*M* = 16.86, *SD* = 3.51) than those in the low threat condition (*M* = 16.53, *SD* = 3.42), *F*(1,368) = 5.87, *p* = 0.016, η*p*^2^ = 0.016. However, there was no significant difference in perceived efficacy between the high (*M* = 18.29, *SD* = 1.93) and low efficacy conditions (*M* = 17.96, *SD* = 2.24), *F*(1,368) = 2.48, *p* = 0.116. As such, ANCOVAs were run on the three efficacy items, to assess if response efficacy or self-efficacy had been successfully manipulated. Both of the response efficacy items did not appear to be affected, *F*(1,368) ≤ 0.32, *p* ≥ *0.5*72. However, those in the high efficacy condition had significant higher self-efficacy (*M* = 6.02, *SD* = 0.94), relative to those in the low efficacy condition (*M* = 5.75, *SD* = 1.20), *F*(1,368) = 6.12, *p* = 0.014, η*p*^2^ = 0.016.

Despite the effective threat manipulation, participants in the high threat condition did not make significantly more health-threat responses (*M* = 4.02, *SD* = 2.11) than those in the low threat condition (*M* = 4.10, *SD* = 2.01), *F*(1,367) = 0.03, *p* = 0.867. Likewise, participants who read a highly threatening article did not have significantly greater intentions (*M* = 57.99, *SD* = 9.33), compared to those who read a less threatening article (*M* = 58.12, *SD* = 9.36), *F*(1,365) = 0.34, *p* = 0.563. Furthermore, intentions were not significantly stronger after reading a high efficacy article (*M* = 58.03, *SD* = 8.90), relative to a low-efficacy article (*M* = 58.09, *SD* = 9.78), *F*(1,365) < 0.01, *p* = 0.967. Contrary to the predictions of the EPPM, there was no significant interaction effect, *F*(1,365) = 0.18, *p* = 0.671. See the electronic supplementary material for correlational analyses.

### Post Hoc* Bayesian Analyses*

As frequentist statistics cannot provide evidence in favour of the null hypothesis [61, 62], we ran a Bayesian ANCOVA on behavioural intentions, using JASP [63]. Results were interpreted according to Jeffreys’ [64] grades of evidence. There was moderate to extreme evidence in favour of the null over the alternative models, BF_10_ = 0.133–0.003, indicating that the data were 7.5 to 333 times more likely under the null model than the alternative models.

## Discussion

This research evaluated the effectiveness of fear appeals in increasing intentions to adhere to COVID-19 mitigation measures. Reading a highly threatening article consistently increased COVID-related threat, indicating that even though participants had been undoubtedly exposed to considerable news about COVID, the manipulation was successful. Nevertheless, contrary to our first hypothesis, a threatening article did not generate stronger intentions to adhere to mitigation measures, across any of the studies. Reading a high efficacy article enhanced self-efficacy, but not response efficacy. Hypothesis 2 predicted that the efficacy manipulation would result in stronger intentions, but that hypothesis was not supported. The EPPM argues that threat campaigns are only effective when paired with higher efficacy. As such, hypothesis 3 expected an interaction between threat and efficacy. However, the interaction (threat × efficacy) also failed to increase intentions. We can be at least moderately confident in this conclusion that neither the threat manipulation nor the efficacy manipulation changed intentions (i.e. hypotheses 1–3 are incorrect) given that the Bayesian analyses conducted robustly supported the null hypothesis. Nevertheless, these results did consistently support hypothesis 4 in relation to COVID-related threat, but not interpretations. The highly threatening articles reliably increased COVID-related threat across studies despite failing to change intentions.

These findings are consistent with Heffner et al.’s 2020 study [43], which similarly found that threatening health messages about COVID-19 were no more beneficial in enhancing intentions to perform mitigation measures relative to messages which strengthened perceived efficacy. However, as our efficacy manipulation did not increase response efficacy, our findings are somewhat aligned with the predictions of the EPPM—which is that in the absence of high response and self-efficacy, threatening messages will be ineffective. Yet, in contrast to the EPPM, there was no evidence of rejection of the message either, as intentions were not lower in the high threat group relative to the low threat group. Unfortunately, we did not measure defensiveness, per se, in the current study and therefore we cannot exclude the fact that defensiveness contributed to null findings. Clearly, Heffner’s results and our findings differ from the predictions of the EPPM and previous research on fear appeals, which have suggested that enhancing threat [16–19], or efficacy [38, 39], would evoke positive changes in health-related intentions, but *why*?

Unlike the more distal health threats (e.g. smoking), which have been the target of previous research, COVID-19 is a highly relevant and immediate health risk. At the time of the first pilot study, little was known about the virus and there had been an influx of threatening news, to which participants were likely exposed. Consequently, participants may have been extremely concerned about COVID-19 already. Indeed, adherence at baseline was already high in both the first pilot study (*M* = 53.28, *SD* = 8.30) and the main study (*M* = 50.70, *SD* = 9.36), which may have been the result of heightened threat. It is worthwhile noting that our findings are inconsistent with research conducted during the 2003 SARS outbreak [20–22] and the 2009 pandemic of H1N1 (swine flu) [23–25]. Whilst this may be surprising, it is also the case that even early on, it seemed clear that COVID-19 would ultimately be a greater threat than H1N1 or SARS. For example, unlike H1N1 or SARS, COVID-19 prompted the introduction of mandatory lockdowns and restrictions across Australia, as in many jurisdictions, which likely created greater adherence to mitigation measures. A high rate of adherence to these measures could explain why a threatening article failed to increase intentions in the first pilot study and the main study. However, adherence was considerably lower at baseline in the second pilot study (*M* = 31.67, *SD* = 10.21) and intentions remained unaffected by the threatening article. Thus, across periods of varying levels of community transmission and restrictions, threatening health messages failed to enhance intentions to perform mitigation measures.

Another objective of this research was assessing the impact of fear appeals upon interpretation bias. The COVID-19 pandemic has resulted in increased levels of anxiety, and interpretation bias was a plausible mechanism through which the threatening information could increase anxiety. In the first pilot study, we found a greater health-threat interpretation bias in the threatening group compared to the reassuring group. However, we were unable to replicate this finding and although interpretation bias did change in study 1, it was not associated with threat or adherence. It is possible that the context of our study affected the results. The first pilot study was undertaken during the middle of May, when there was more uncertainty about COVID, and restrictions were currently in place. In comparison, the following studies were conducted in November and July—when restrictions in Australia were more relaxed. This easing of restrictions, following months of low levels of community transmission, may have instilled a sense of normalcy amongst participants. In these less concerning times, the threatening information may have been less likely to impact how individuals interpreted ambiguous information. Similarly, by July and November, participants would have been exposed to more information about COVID, with which the current messages had to compete. Consistent with these explanations, the threat manipulation constituted a large effect size in the first study, whilst it only generated smaller effects in the later studies. It seems likely that a more robust threat manipulation may be needed to induce interpretation biases.

## Limitations

There were some limitations that should be borne in mind when interpreting the results. Firstly, as Australia did not experience high levels of community transmission of COVID-19 in 2020, these findings may not generalise to more affected countries. Secondly, the generalisability of our findings in study 3 was limited by the female-only sample and the difficulties we faced recruiting a gender diverse sample of participants through social media. It is unclear why we had these difficulties; however, previous research has suggested that females are more willing to participate in research [65, 66]

We also want to acknowledge that our article manipulations may have been limited in their influence as they were competing with considerable COVID-related media at the time. Whilst this may have limited the impact of our manipulation, participants in all three studies reported feeling more threatened by COVID after reading the threatening article, suggesting that if threat was sufficient to change intentions, we should have seen an impact. In addition to this, in our second pilot study, those who read the threatening COVID article also were able to correctly recognise a greater proportion of COVID-related facts, relative to those who read a control article. Thus, even though there was an oversaturation of competing messages about COVID-19, our articles did contain new information and led people to feel more concerned and threatened by COVID.

Lastly, although using intentions as a proxy for behaviours is common, intentions are not an infallible predictor of behaviour [67]. Hence, we cannot conclude that the intentions expressed by participants were acted on. However, we should note that adherence to mitigation measures at baseline correlated highly with intentions in all studies (*r* ≥ 0.74). Nevertheless, to confirm this, future research should implement follow-up measures of behaviours.

### Implications

Although our articles did not affect intentions, they were successful in manipulating COVID-related threat and self-efficacy. Crucially, if a single article—amongst the media consumed daily—affects these appraisals, then it is worthwhile considering what the cumulative impact of exposure to alarmist articles may have over time. Whilst future research is needed to determine the impact of repeated threatening articles, our results suggest that more reassuring, yet still informative health messages, do not compromise intentions to adhere to mitigation measures and result in fewer COVID-related concerns. Although we cannot be sure that these intentions would translate into sustained adherence in the real world, it is clear that there is a difficult balance which must be struck between providing informative news and causing unnecessary fear.

### Electronic supplementary material

Below is the link to the electronic supplementary material.

Supplementary file1 (DOCX 25 KB)
Supplementary file2 (DOCX 809 KB)
