# Supplementary material for: Are Fear Campaigns Effective for Increasing Adherence to COVID-Related Mitigation Measures?
Source: Int J Behav Med. 2022 Nov 1;30(5):714–30. doi: 10.1007/s12529-022-10137-y (PMC9628502; doi:10.1007/s12529-022-10137-y)
Supplement: Supplementary file 1 — Supplementary file1 (DOCX 25 KB) Supplementary file2 (DOCX 809 KB) [file 12529_2022_10137_MOESM1_ESM.docx]

**Supplementary Materials**

**Table 1**

*Threat Manipulation Examples*

| Article Fact | High Threat Frame | Low Threat Frame |
| --- | --- | --- |
| The death rate ranges from 4-10% depending on source. | “The rate of death…has now reached 4% globally according to the World Health organisation, and 5% in the UK and nearly 10% in Italy.” | “…these rates are *likely inflated* due to the fact that most people who are infected experience a mild case which may go unreported.” |
| 20% of COVID-19 cases are severe. | “So far around 20 per cent of COVID-19 cases have been classified as “severe”, which is *no small amount* considering *half a million* cases worldwide.” | “In fact, 80% of COVID-19 cases *are mild* and *don’t require* any medical attention at all.” |
| In China only 1 per cent of the people who died were otherwise healthy | “This one percent is still a *large number…* if it infects 1 million healthy people…10,000 healthy individuals are *expected to die*.” | “*Only* 1 per cent of the people who died were otherwise healthy [in China]". |
| There have been 97 deaths in Australia | “We have seen already 97 deaths in Australia and many commentators wonder how *many more* we will experience when those recently diagnosed get r*eally sick*.” | “It is also really good news that we have *only* had 97 deaths, which is a *very low proportio*n (only 4 per million Australians).” |

**Table 2**

*Efficacy Manipulation Examples*

| Information | High Efficacy Frame | Low Efficacy Frame |
| --- | --- | --- |
| The overall effectiveness of mitigation measures | “The introduction of these measures, and the dedication of the Australian public to abide by the restrictions and recommendations, was *responsible* for the *successful* flattening of the curve of infection” | “Although, some are claiming that these measures were responsible for flattening the curve…the rate of community transmission was very low already, meaning these measures may have been *completely unnecessary*.” |
| The ease of social distancing | “Thankfully, with advances in technology socially distancing is much *easier*.” | “Social distancing has taken a *large toll* on the mental health of many who *struggle* with social isolation.” |
| The impact on the workforce | “For example, video conference technology, such as zoom, allows us to…work from home” | “Whilst, numerous businesses have had to shut down for *many months*” |

**Table 3**

*Correlational Analyses*

|  | COVID-related anxiety | Death Anxiety | Past Adherence | Attitudes | Subjective Norms | COVID-related Threat | Self-Efficacy | Interpretation Bias | Behavioural Intentions |
| --- | --- | --- | --- | --- | --- | --- | --- | --- | --- |
| COVID-related Anxiety | - | **.31**** | **.39**** | **.43**** | **-.15**** | **.52**** | **-.15**** | **.11*** | **.40**** |
| Death Anxiety |  | - | .02 | .09 | **-.12*** | **.23**** | -.08 | **.11*** | .06 |
| Past Adherence |  |  | - | **.70**** | **.17**** | **.35**** | **.12*** | -.05 | **.80**** |
| Attitudes |  |  |  | - | .07 | **.46**** | .07 | .01 | **.80**** |
| Subjective Norms |  |  |  |  | - | -.06 | **.20**** | -.03 | **.18**** |
| COVID-related Threat |  |  |  |  |  | - | **-.13*** | .04 | **.43**** |
| Self-Efficacy |  |  |  |  |  |  | - | -.07 | **.21**** |
| Interpretation Bias |  |  |  |  |  |  |  | - | -.02 |
| Behavioural Intentions |  |  |  |  |  |  |  |  | - |

**p* < .05

***p* < .01
